# Honey bee (*Apis mellifera*) exposomes and dysregulated metabolic pathways associated with *Nosema ceranae* infection

**DOI:** 10.1371/journal.pone.0213249

**Published:** 2019-03-07

**Authors:** Robert L. Broadrup, Christopher Mayack, Sassicaia J. Schick, Elizabeth J. Eppley, Helen K. White, Anthony Macherone

**Affiliations:** 1 Department of Chemistry, Haverford College, Haverford, PA, United States of America; 2 Department of Biology, Swarthmore College, Swarthmore, PA, United States of America; 3 Molecular Biology, Genetics, and Bioengineering, Faculty of Engineering and Natural Sciences, Sabancı University, İstanbul, Turkey; 4 Life Science and Chemical Analysis Group, Agilent Technologies, Santa Clara, CA, United States of America; 5 Department of Biological Chemistry, The Johns Hopkins University School of Medicine, Baltimore, MD, United States of America; University of British Columbia, CANADA

## Abstract

Honey bee (*Apis mellifera*) health has been severely impacted by multiple environmental stressors including parasitic infection, pesticide exposure, and poor nutrition. The decline in bee health is therefore a complex multifactorial problem which requires a holistic investigative approach. Within the exposome paradigm, the combined exposure to the environment, drugs, food, and individuals’ internal biochemistry affects health in positive and negative ways. In the context of the exposome, honey bee hive infection with parasites such as *Nosema ceranae* is also a form of environmental exposure. In this study, we hypothesized that exposure to xenobiotic pesticides and other environmental chemicals increases susceptibility to *N*. *ceranae* infection upon incidental exposure to the parasite. We further queried whether these exposures could be linked to changes in conserved metabolic biological pathways. From 30 hives sampled across 10 sites, a total of 2,352 chemical features were found via gas chromatography-time of flight mass spectrometry (GC-TOF) in extracts of honey bees collected from each hive. Of these, 20 pesticides were identified and annotated, and found to be significantly associated with *N*. *ceranae* infection. We further determined that infected hives were linked to a greater number of xenobiotic exposures, and the relative concentration of the exposures were not linked to the presence of a *N*. *ceranae* infection. In the exposome profiles of the bees, we also found chemicals inherent to known biological metabolic pathways of *Apis mellifera* and identified 9 dysregulated pathways. These findings have led us to posit that for hives exposed to similar chemicals, those that incur multiple, simultaneous xenobiotic stressors have a greater incidence of infection with *N*. *ceranae*. Mechanistically, our results suggests the overwhelming nature of these exposures negatively affects the biological functioning of the bee, and could explain how the decline in bee populations is associated with pesticide exposures.

## Introduction

Bees are essential to maintaining biodiversity, and their services as crop pollinators cannot be overstated[1–5]. Over the past decade global honey bee populations have been severely reduced by various environmental stressors including but not limited to: poor nutrition, losses in foraging habitats, infectious exposures to viruses and parasites, and exposures to pesticides and other persistent chemicals[6–9]. Each of these individual stressors represents environmental exposures that adversely affect bee health at the colony level. Moreover, these environmental exposures may interact with one another and synergistically effect the overall health of the hive[10–12]. One study indicated that the interaction between viral infections and the parasitic mite, *Varroa destructor*, is a primary cause of honey bee colony mortality[13]. In another study, the probability of infection by the fungus, *Nosema spp*., has also been found to be substantially increased when bees are concomitantly exposed to fungicides[14, 15]. Finally, *Nosema spp*. infection coupled with exposure to the neonicotinoid pesticide imidacloprid has been observed to weaken the ability of honey bees to sterilize the colony and brood food, rendering the hive more susceptible to pathogens[11]. These studies suggest that there is a complex relationship between environmental stressors and hive survivability, implying that a more holistic approach is needed to determine how potential causal factors interact to result in bee health decline.

The exposome paradigm considers the life-time sum of environmental exposures starting from conception onwards. Rooted in the field of cancer epidemiology, the exposome was espoused to account for the environmental contributions to disease onset and progression, and is woven upon an inherited genetic framework–the combination of which contributes to the phenotypic variation of biological traits[16]. The exposome paradigm utilizes “omic” (big data) technologies for the characterization of environmental exposures and constitutes measuring both the external (air pollution, pesticide exposure, xenobiotics, etc.) and internal chemical environment (metabolism, activity of the microbiome, oxidative stress, etc.) of the organism. How these exposures might result in changes to the internal environment (metabolome)—governed by highly conserved biological pathways—is also considered, as is how they might serve as chemical biomarkers of changes in the phenotype[17].

*Nosema ceranae* is a fungal gut pathogen that is distributed worldwide[18, 19]. The rise in prevalence of this fungal gut pathogen is thought to be facilitated by sub-lethal exposure to pesticides causing suppression of the immune system[20]. *Nosema ceranae* is thought to be replacing *Nosema apis* throughout much of the world in its role in the decline of been health[21–23]. On its own, *N*. *ceranae* is suspected to be a benign chronic infection on the individual and colony level in comparison to common honey bee viral and bacterial infections, but it still causes immune suppression, energetic stress, and malnutrition, eventually shortening the life-span of the honey bee[24–30]. Therefore, in combination with other stressors such as pesticides and other infectious exposures, there can be a synergistic increase in mortality[10, 11].

The mode of action of many fungicides and herbicides typically target highly conserved biological pathways; consequently, there is a potential for detrimental non-target effects in other organisms containing the same highly conserved pathways. Therefore, it is possible that simultaneous exposure to *N*. *ceranae* and multiple pesticides may impact critical metabolic pathways, rendering a decline in immune function or other biological activity[30–33]. However, from a mechanistic point of view, the manner in which multiple, simultaneous exposures affect honey bees’ metabolic pathways remains largely unknown.

Traynor, et al. (2016) used a targeted exposome approach and focused on pesticide exposures to determine associations between total pesticide burden and hive mortality[34]. These authors further explored the influence of pesticide exposures on re-queening events by measuring the difference in the total number of pesticides at the beginning and end of the study. However, except for modes of action, no specific biological response (pathways) information was presented.

**Fig 1:**
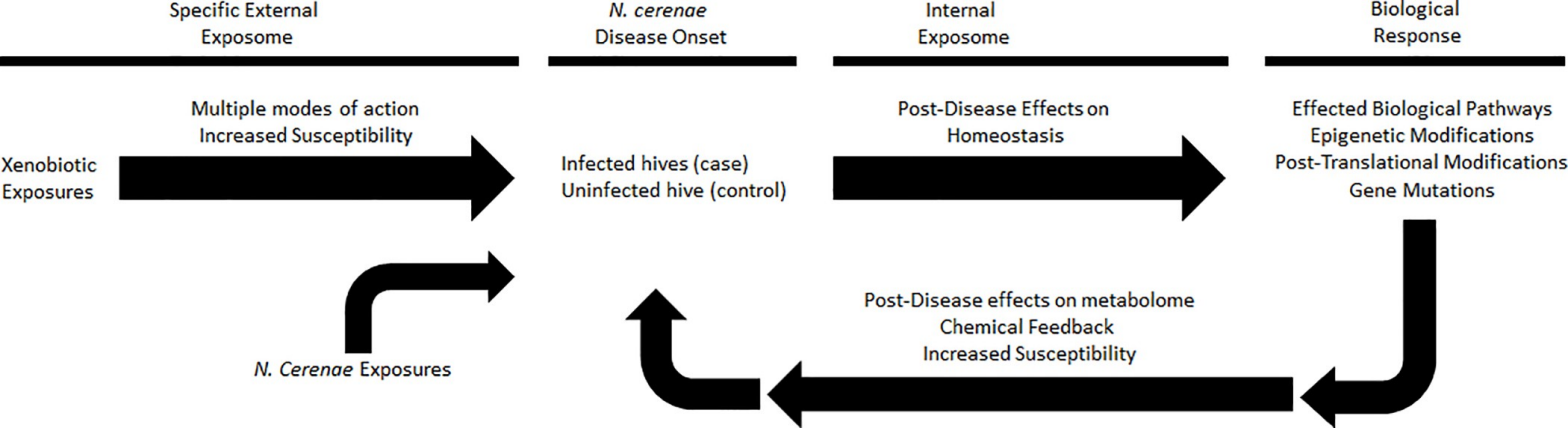
Overview of integrating the effects of disease and environmental exposures. Along the pre-disease causal pathway, specific external exposures affect measurable chemical changes and increase susceptibility to disease through multiple Modes of Action (MOAs). These exposures affect biological pathways, change the metabolome, and influence genetic expression, transcription, and post-translational and epigenetic modifications. Along the post-disease reactive pathway, measurable chemical changes in the various “omes” feedback and can further increase disease susceptibility in an increasingly adverse feedback loop.

In our study, we demonstrate how exposomics can be used in a comprehensive manner that integrates targeted disease screening of a pathogens (*N*. *ceranae*) with colony level honey bee exposome profiles to seek out novel associations between exposures and disease pathology. Using the exposomic approach to account for environmental exposures like pesticides and *N*. *ceranae* infection as an indicator of health status, we can begin to unravel the intricacies of how these stressors can potentially interact mechanistically. Our intent is to shed light on how exposed bee hives may become even more susceptible to disease or how diseased colonies may be more susceptible to chemical exposures, which may lead to an increase in colony mortality as a result of multiple exposure events (for details see Fig 1).

## Methods

### Sample collection

Adult foraging bees were sampled once during the 2015 season from 30 hives in 7 different geographical locations in the southeastern Pennsylvania region. The locations were selected to represent urban and suburban settings (S4 Table). Sixty to one hundred bees in total were collected from each hive in 50 mL disposable tubes and immediately frozen on dry ice. All samples were then stored in a -80°C freezer. Prior to analysis the samples from each hive were gently thawed and randomly divided into two groups: one group for semi quantitative-PCR, and a second group for extraction and sample cleanup prior to GC-TOF analysis. Each of the resulting 60 samples (30 hives x 2 groups/hive) represent a snapshot of the parasitic load and exposome profile of the hive from which it was collected at that specific time. All samples were randomized prior to analysis.

### Semi-quantitative-PCR screening for *Nosema spp*

*DNA Extraction*: Pooled samples for semi-quantitative PCR analyses were prepared by adding 6 mL of DNase and RNase free water to 30 bees collected from each hive. The bees were thoroughly homogenized with a sterile 50 mL tissue grinder (Fisher Scientific). A total of 300 μl of an extration buffer (0.03 M hexadecyltrimethyl ammonium bromide, 0.05 M tris hydroxymethyl aminomethane, 0.01 M ethylenediamine tetra-acetic acid, and 1.1 M NaCl, in d H2O) was added to a 150 μL aliquot of bee homogenate which was then macerated further using a sterile pestle. Then 4 ul of Proteinase K solution (20 mg/ml) was added to the macerated sample and incubated at 60°C on a heat block for 3 hr. Following this, the samples were placed on ice and 300 μL of a 1:1 mixture of phenol/chloroform was added to the 1.5 ml microcentrifuge tube. The solution was centrifuged at 13,000 rpm for 5 min, and the supernatant was added to another 300 μL of the 1:1 phenol/chloroform solution in a new 1.5 mL microcentrifuge tube. This supernatant was then added to 300 μl of chlorofom where the supernatant was drawn once again and transferred to a new 1.5 mL microcentrifuge tube that was then followed by the addition of 30 μL sodium acetate (3 M) and 600 μL 95% ethanol to precipitate the DNA overnight at -20°C. For the PCR reaction, the extracted DNA from each sample was quantified using a Nanodrop 2000 UV-Vis Spectrophotometer (Thermo Scientific). Each sample was then diluted with DNase-free water to a working concentration of 5 ng/μl to serve as the PCR template DNA according to the HBRC method [35].

*Semi-quantitative-PCR*: A 15 μL duplex PCR reaction (one for *N*. *ceranae* and one for *N*. *apis*) combined: 1 μL of a 10 mM solution of each of the four primers (4 μL total volume); 1.5 μL of 10x PCR buffer; 0.5 μL of 10 mM deoxynucleotide triphosphate (dNTP); 0.2 μl of a 25 mM magnesium chloride solution; 0.2 μL of 5 U/μL Taq DNA polymerase (New England BioLabs, Ipswich, MA) with 2 μL of template DNA from the DNA extraction described above and 6.6 μL Millipore water (0.2 μm sterile filter). Specific primers that identify and distinguish *N*. *apis* and *N*. *ceranae* based on a unique sequence found in a highly conserved ribosomal gene were used. To semi-quantify both *N*. *ceranae* and *N*. *apis*, a PCR multiplex reaction with a *RpS5* reference gene (S5 Table) was performed [35]. The PCR thermocycler program was 94°C for 2.5 min, followed by 10 cycles of 15s at 94°C, 30s at 61.8°C and 45s at 72°C, and 20 cycles of 15s at 94°C, 30s at 61.8°C and 50s at 72°C, an extension step at 72°C for 7 min, and a final hold step of 4°C. Each PCR assay included a negative control containing no DNA template and a positive control which was shown to be positive in an infected sample in previous PCR runs, for each *Nosema* species. PCR products were confirmed by a 3% gel electrophoresis at 100 v for 3.5 hours with a low molecular weight DNA ladder (New England BioLabs, Ipswich, MA). Each sample was run in triplicate and the relative amount of DNA was determined using densitometry and Image J software[36]. Values were averaged across the triplicates and divided by the *Apis mellifera RpS5* reference gene to calculate the relative abundance of *N*. *ceranae* or *N*. *Apis* as densitometric values and were also presented as spores per bee in the results for each hive sampled.

### Honey bee Extraction for GC-TOF analysis

Honey bees were extracted using previously published QuEChERS extraction protocols[37]. Briefly, 3 grams of foraging bees (~30 bees) were pulverized in 27 mL 44:55:1 water/acetonitrile/acetic acid and transferred to 50 mL disposable tubes. Six grams of magnesium sulfate and 1.5 g sodium acetate were added to the suspension, and the tubes were sealed, shaken, and thoroughly mixed before centrifugation at 3000 rpm for 5 minutes. A 2-mL aliquot of the supernatant from each sample was applied to conditioned C_18_ SPE cartridges (Agilent Technologies, Santa Clara, CA). The analytes were eluted with a 70:30 solution of acetone/toluene and reduced in volume using a Savant Speed Vac, SC110, before transfer to 2 mL auto-sampler vials prior to GC-TOF analysis.

### GC-TOF

For discovery-based (non-targeted) exposome profiling of honey bee extracts, an Agilent 7890B/7200B gas chromatography-quadrupole time of flight mass spectrometer (GC—QTOF) system was used. A 0.2 μL pulsed split-less injection was made into a 250°C isothermal split/split-less inlet. The GC was configured with a 40 m x 0.25 mm x 0.25 μm DB5-MS DuraGuard column (J&W 122-5532G) operated at 1.2 mL/minute helium in constant flow mode. The oven program was 80°C (1 minute) then, 10°C/min to 310°C (6 minutes). The transfer line temperature was 300°C. The mass spectrometer was operated in electron ionization, high resolution TOF mode. The source and quadrupole (RF only) temperatures were 275°C and 150°C, respectively. High resolution, accurate mass (HRAM) spectral data was collected at 5 Hz over a mass range of 50 Da to 800 Da. Automated intra-sequence mass calibration was performed immediately prior to each sample injection. All samples were run in duplicate and the average of the two runs used for data analysis.

### Chromatographic deconvolution and chemical entity annotation

Raw data acquired on the GC-QTOF system was analyzed using the MassHunter suite of software. To this end, Unknowns Analysis B.08.00 was used to perform chromatographic deconvolution of the 60 data files collected in this pilot study (30 samples x 2 injections per sample). Chemical features were minimally identified as having signal-to-noise ratio > 3:1, an accurate mass assignment for the base ion and a retention time for the chromatographic peak where the feature is found.

Spectral library searches and compound annotation were performed using the RTL Pesticides and the Fiehn Metabolomics libraries (Agilent Technologies, Santa Clara, CA) and the NIST-11 Mass Spectral Library (the National Institute of Standards and Technology, NIST Standard Reference Database 1A v11). For the 629 chemical features that were not identified, the minimal feature parameters defined above and a composite mass spectrum was used for covariate statistical analysis.

### Modes of action

We combined the 20 identified xenobiotics into 4 categories: MOAs affecting Na+ or Ca2+ were grouped into an Ionic (Na+, Ca2+) Interference category, MOAs affecting mitosis or microtubule assembly, were grouped into a Physiological Effects category, MOAs affecting acetylcholinesterase inhibition, oxidative phosphorylation inhibition, or interference with ATP production, were grouped into a Chemical / Enzyme Interference category and lastly, we created a Multiple Modes of Action category. We determined the number of exposure events for the infected and uninfected groups for each of the MOA categories.

### Statistical testing and covariate analysis

To identify associations of exposome profiles with *N*. *ceranae* infection, the mass spectrometry datasets collected in this study were statistically analyzed with MassProfiler Professional (MPP) bioinformatics software to identify chemical features associated with the *N*. *ceranae* infected samples. This process entailed aligning the retention times of each compound, establishing a baseline based on the median abundance of all chemical entities detected, and filtering where only samples remained in the dataset that had a relative ion abundance fold-change at least three times the median ion abundance calculated across all samples. This filtered data then underwent significance testing using an unpaired T-test, p < .05 to compare each chemical found in uninfected and infected hives, while correcting for multiple testing and false discovery rates. The chemicals with significant differences that could also be located within the Kyoto Encyclopedia of Genes and Genomes (KEGG) database were mapped onto known *Apis mellifera* biological pathways[38, 39]. Presuming the probability of xenobiotic exposures is binomially distributed in each of the 4 MOA categories, there is an equal probability that an exposure event in each category will affect the onset of *Nosema* or it will not. We calculated p-values at α = .05 to assess the association between the number of exposures and *Nosema* infection in each category. Chi-sqaure xenobiotic burdens associated with infection status and xenobiotic mode of actionanalyses along with graphics generation were performed using Microsoft Excel 2016, R and R Studio software[40].

### Ethics statement

The samples were sourced from 7 private apiaries with the permission of the apiary owners/managers. Therefore, no government approval or licenses were required for their collection. Honey bees are invertebrates so no special ethical approval is required. The honey bees used for this study were euthanized humanely by placement on dry ice immediately after live-sampling from individual hives. The collection and use of such samples were not killed for the sole purpose of this study. The animals collected are not cosidered endangered or protected by any federal agency. All samples were colleted with permission from the following private land owners: Donald Shump and The Philadelphia Beekeepers Guild, Awbury Arboretum, Philadelphia, PA (40.050621, -75.1681528), Carmen Battavio, Carmen’s Bees, West Chester, PA (40.0479805, -75.5400049), Robert L. Broadrup, Four Bees Apiary, Malvern, PA (40.047796, -75.539938), Claudia Kent, Eli St. Amour, Haverford College, Haverford, PA (40.0117552, -75.2994395), Donald Shump and The Philadelphia Bee Co., Mount Moriah Cemetery, Philadelphia, PA (39.9289388, -75.235422), Keith Jardine, SAP, Newtown Square, PA (39.9880211, -75.4153618), Christopher Mayack, Swarthmore College, Swarthmore, PA (39.9069574, -75.351473).

## Results

### *Nosema ceranae* infection load

The *N*. *ceranae* infection data were approximately log-normally distributed. The mean (± s.d.) *Nosema* load was 3.99 (547,045 spores/bee) ± 9.22 (1,324,278 spores/bee), and the median value was 0.54 (34,340 spores/bee), with a range of 0.00 (0 spores/bee) to 40.02 (5,901,482 spores/bee). Based on these data, the samples were divided into infected (*N*. *ceranae* load > 0, n = 18) and uninfected (*N*. *ceranae* load = 0, n = 12) groups, no *N*. *apis* was detected.

### Exposome analysis overview

**Fig 2:**
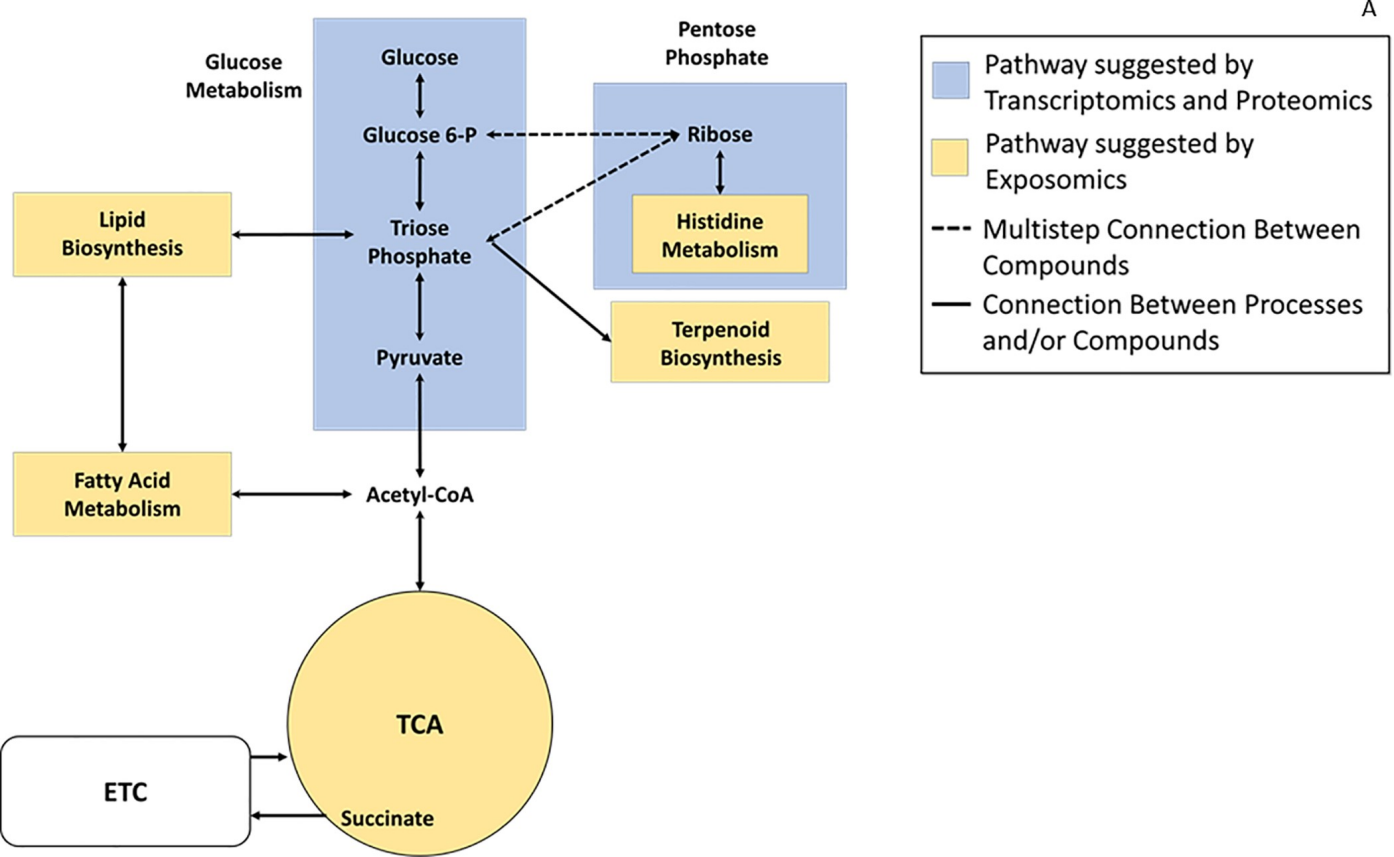
KEGG Pathway analysis. (A) Overview of affected metabolic pathways from a *N*. *ceranae* infection that have been determined from previous transcriptomic and proteomic analysis[30, 32, 33] and additional new pathways affected suggested by significant differences in metabolite relative abundances from the exposomic analysis. TCA represents The Citric Acid Cycle while the ETC represents the Electron Transport Chain. (B) Location of the 14 significantly higher or lower relative abundance compounds mapped onto 9 corresponding *Apis mellifera* metabolic pathways using the KEGG database. Compounds with significantly lower relative abundances are denoted with a red dot while chemicals with significantly higher abundances are denoted with a green dot. The pathways potentially affected are shown with different colors and their immediate connections revealed by comparing uninfected and uninfected hives are marked in red. Each color denotes a different metabolic pathway that contains a corresponding label. Other chemical names in the pathways are not labeled and less connected metabolic pathways have been removed for clarity. Grey boxes represent metabolic pathways not directly linked to a change in metabolite relative abundance in *N*. *ceranae* infected hives. (C) The difference of the means in fold changes (log 2) for the significant 14 chemical relative abundances identified between *N*. *ceranae* infected and uninfected hives corresponding to the nine KEGG pathways shown in Fig 2B. Error bars represent standard deviation of the difference between *N*. *ceranae* uninfected and infected chemical abundances.

After chromatographic deconvolution, we identified a total of 2,352 chemical features with a signal to noise ratio of ≥ 3:1. Of the 2,352 identified chemical features, 1,723 (73%) were annotated (retention time, ion abundance, m/z, chemical name, CAS number) by spectral library search. Twenty known xenobiotics were also identified and were consistently found in each hive (Table 1). These were categorized by mode of action (MOA) into four broad categories (S3 Table). Twenty xenobiotics were observed a total of 143 times across all thirty samples. Each sample contained at least one xenobiotic, and the maximum number of xenobiotics identified in a single sample was 8. The average number of xenobiotics identified per hive was 5. Using this differentially expressed chemical entity sub-set of biological and naturally occurring chemicals in the bee exposome profiles, we further identified a total of 14 compounds associated with 9 honey bee metabolic pathways, through pathways analyses, we determined the relative higher or lower abundance of these compounds (Fig 2; Table 2).

**Table 1:** Xenobiotics identified in honey bee exposomes.

*Compound*	*Category*	*CAS Number*
*Praziquantel*	Antiparasitic	55268-74-1
*Captafol*	Fungicide	2425-06-01
*Captan*	Fungicide	133-06-2
*Fenpropimorph*	Fungicide	67564-91-4
*Propamocarb*	Fungicide	25606-41-1
*Carbetamide*	Herbicide	16118-49-3
*Benzoylprop-ethyl*	Herbicide	22212-55-1
*Tebutam*	Herbicide	35256-85-0
*Allethrin*	Insecticide	584-79-2
*Azobenzene*	Insecticide	103-33-3
*Carbofuran*	Insecticide	1563-66-2
*Ethiofencarb*	Insecticide	29973-13-5
*Flurecol-butyl*	Insecticide	2314-09-2
*Isoprocarb*	Insecticide	2631-40-5
*Monocotophos*	Insecticide	919-44-8
*Promecarb*	Insecticide	2631-37-1
*Propoxur*	Insecticide	114-26-1
*Tetramethrin*	Insecticide	7696-12-0
*Oxalic Acid*	Varroacide	6153-56-6
*Naphthalene*	Polycyclic aromatic hydrocarbon	91-20-3

Chemical category and CAS number provided for each xenobiotic.

**Table 2:** Broad MOA categories.

*Broad MOA Categories*	*n*	*# Infected*	*# Uninfected*	*p*	*-Log*_*10*_*p*
*Ionic (Na+*, *Ca*^*2+*^*)interference*	81	50	31	0.0096	2.02
*Chemical / enzyme interference*	15	11	4	0.042	1.4
*Multiple modes of interaction*	10	7	3	0.12	0.93
*Pysiological effects*	6	2	4	0.23	0.63

The total number of exposure events (n) in each category, the number of exposure events stratified by infected and uninfected and, p-values and -Log10 p-values determined for each.

### Xenobiotic burden and *N*. *ceranae* infection

**Fig 3:**
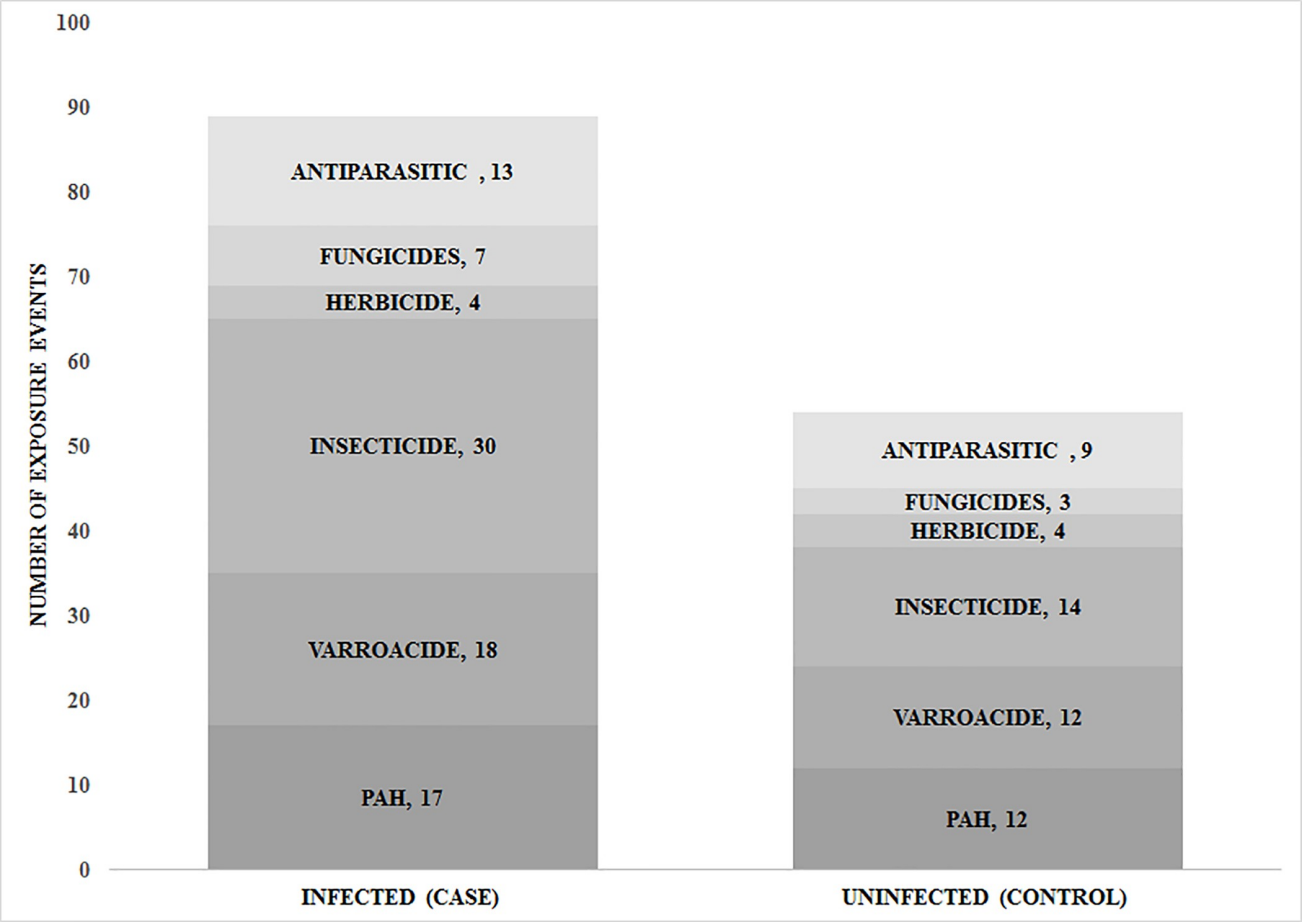
Cumulative number of xenobiotic exposure events. Xenobiotics identified in exposome profiles of honey bee foragers categorized by *N*. *ceranae* infected and uninfected hives. Data represent the total number of exposure events grouped by each class of xenobiotic for both the Infected and Uninfected hives as determined by presence or absence of *N*. *ceranae* infection. Insecticides accounted for 32%, varroacides (oxalic acid) 21%, PAH (naphthalene) 20%, anti-parasitics 14%, fungicides 7% and herbicides 6% of the exposures. Infected hives have significantly more exposure events as compared to uninfected hives (χ2 = 7.619, df = 1, n = 30, p < 0.006).

Out of the 20 identified xenobiotics, 18 were found in the *N*. *ceranae* infected hives, while only 10 out of 20 were found in uninfected hives. Additionally, the total number of xenobiotics exposure events were significantly higher in *N*. *ceranae* infected colonies (χ2 = 7.619, df = 1, n = 30, p < 0.006) (Fig 3). However, the relative levels of these exposures were not associated with *Nosema* infection load (χ2 = 0.168, df = 1, n = 30, p = 0.682, S1 Table). Even when grouped by chemical category, there was no association between the relative level of exposure and *Nosema* infection load (S1 Fig and S1 Table).

### Xenobiotic mode of action (MOA)

**Fig 4:**
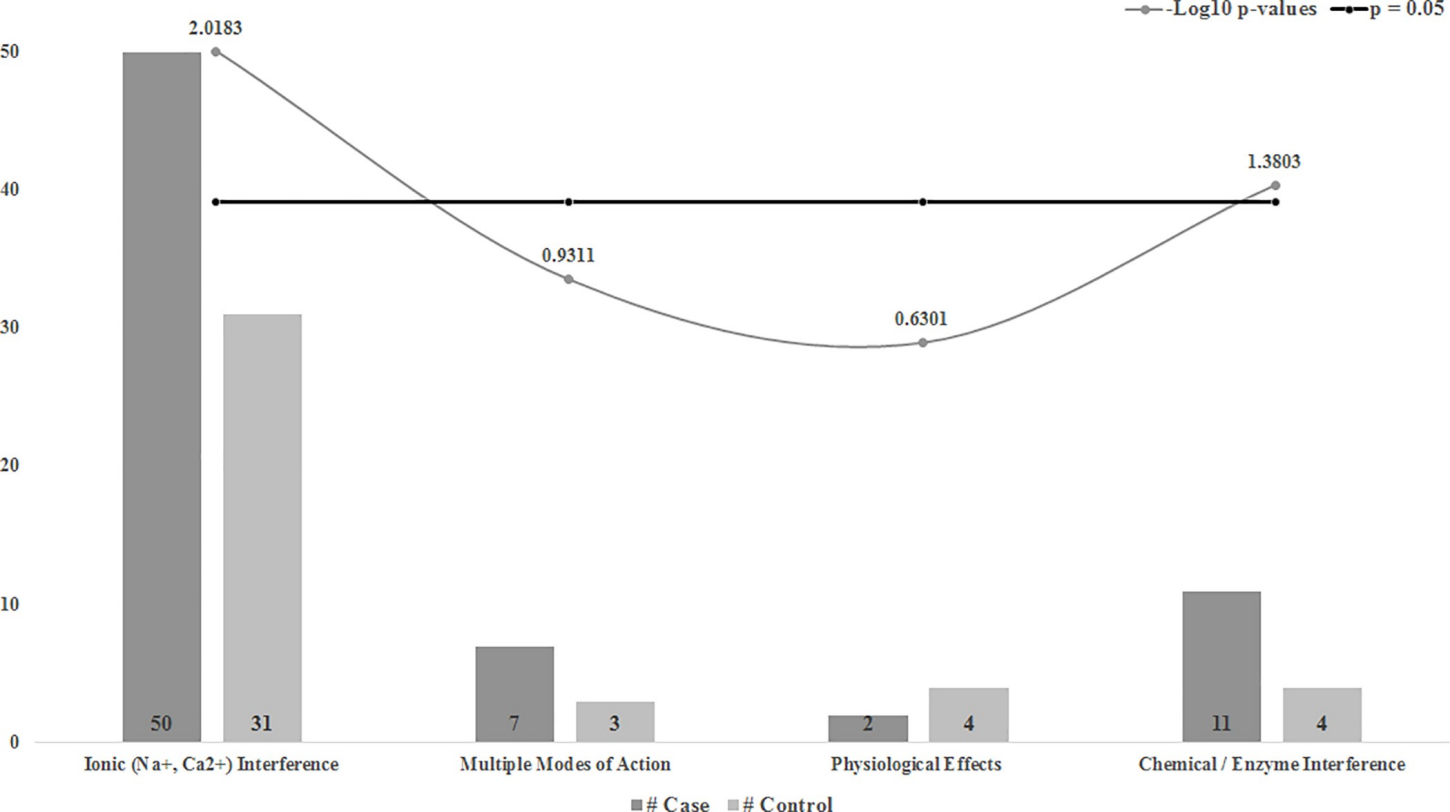
Broad categories of chemical exposures for infected and uninfected groups. Data represents the number of exposure events grouped into one of four modes of action with each bar representing either Infected (dark bar) or Uninfected (light bar) hives side by side for comparison. The p-value for each category is given in the superimposed curve and the p = 0.05 cut-off is represented by the black horizontal line. P-values are -Log10 transformed, so any value above this black line is significant and anything below it is non-significant.

We combined the MOA categories into four broad mechanistic categories that revealed a total of 112 xenobiotic exposure events. Infected hives were about 65% more exposed to different xenobiotic MOAs when compared to uninfected hives. More specifically, there were higher xenobiotic exposures classified with an Ionic (Na+, Ca2+) and Chemical/Enzyme Interference MOA categories in *N*. *ceranae* infected hives (p < .01, and p < .05, respectively (Fig 4) (Table 2)).

### KEGG pathway analysis of metabolites

In *N*. *ceranae* infected bees, we found that out of the 14 annotated chemicals that could be mapped onto KEGG pathways, succinate was located in the citric acid (TCA) cycle, naphthalene elicits the activation of the p450 detoxification pathway, beta-tocopherol was located within the ubiquinone and other terpenoid-quinone biosynthesis pathway, 3,4-dihydroxyphenylethylene glycol was found in the tyrosine metabolism pathway, dimethyl sulfone was part of the sulfur metabolism pathway, squalene was located in the terpenoid biosynthesis pathway, beta-sitosterol within the steroid biosynthesis pathway, 4-(beta-acetylaminoethyl) imidazole in the histidine metabolism pathway, and both (9Z)-octadecenoic acid and octadecanoic acid were end products of the fatty acid biosynthesis pathway. These were found to have a significantly higher relative abundance in *N*. *ceranae* infected hives. In contrast, sulfur (cyclic octaatomic sulfur) part of the sulfur metabolic pathway, isofucosterol part of the steroid biosynthesis pathway, N (pi)-methyl-L-histidine within the histidine metabolism pathway, and hexadeconic acid linking the fatty acid degradation and biosynthesis pathways had significantly lower abundances in *N*. *ceranae* infected hives. Each metabolic pathway affected by multiple metabolite dysregulation did not contain consistent higher or lower relative abundances (Fig 2).

## Discussion

### Xenobiotic exposures associated with *N*. *ceranae* infection

Our results support the notion that it is not the amount of pesticide exposure, nor a particular kind of pesticide exposure, but rather the number of exposure events from different xenobiotics that is associated with *N*. *ceranae* infected hives. In most previous lab studies, only one pesticide at a time was tested, and from these it has been suggested that pesticide exposure suppresses the honey bee immune system, which increases the risk of contracting an infection[10, 11, 31]. However, we did not find an association with a *N*. *ceranae* infection from the amount or number of exposure events from any one pesticide in particular. This is important to note because it supports the idea that multiple pesticides at sub-lethal levels can still interact with one another to produce synergistic declines in bee health[41, 42]. This may be particularly the case in honey bees where it is suggested that they have a limited capability to detoxify xenobiotic exposures in comparison to less social insects[43]. These increased expsoure events are likely to result in increased pathogen susceptability[44, 45]. Our findings highlight that honey bees are regularly subjected to a number of pesticides because twenty chemicals were observed a total of 143 times across all thirty hives. The majority of the xenobiotics identified are not known to be used in agricultural settings. Relatively little is known about how multiple pesticide residue exposures impact the life stages of honey bees or wild pollinators, and our study supports the notion that routes of pesticide exposure outside of the crop field should be considered when considering susceptibility to *N*. *ceranae* infections[46].

Even though both *Nosema* infected and uninfected hives were exposed to similar xenobiotic chemicals, and both groups contained xenobiotics representing all 6 chemical categories, the fact that we find no particular type of xenobiotic associated with a *N*. *ceranae* infection is somewhat surprising because neonicotinoids tend to be the focus of many bee health studies[47], and fungicide exposure has been the focus of increased *N*. *ceranae* infection susceptibility[46]. Here, collectively, we identified that insecticides (30 across the infected group, 14 across the uninfected group) and fungicide exposures (7 across the infected group, 3 across the uninfected group) make up less than half of the exposure events in our dataset. These findings agree with a recent study that demonstrated how bees sampled from the field are more likely to be exposed to a range of pesticides encountered through diverse routes of exposure in contrast to the neonicotinoids which dominate focal crop pollen foraging[48].

We consistently detected older classes of pesticides such as carbamates, pyrethroids, and organophosphates. Additionally, the xenobiotics identified in our study differ somewhat from those reported in previous studies[37]. For example, we did not detect coumaphos, a common insecticide with known acetylcholinesterase inhibition activity, in the foraging bees. This may reflect our sample set being comprised of hives from beekeepers located in suburban and urban areas as opposed to commercial hives that are often transported for pollination services in more rural locations.

### Xenobiotic mode of action (MOA)

We found various xenobiotic exposures with different modes of action (MOA), which gives us insight into the biological functional implications of the xenobiotic exposure. A disproportionate amount of sodium and calcium can affect many physiological processes ranging from osmotic balance to the functioning of the central nervous system. The significantly higher amount of exposures with ionic interference may be due to the disproportionate amount of exposure to insecticides found with *N*. *ceranae* infected bees, many of which—such as neonicotinoids—are known to disrupt acetylcholine activity in the bee brain. The greater number of exposures that interfere with enzymes can be likely attributed to the fungicides and herbicides, which are identified to disrupt enzymes involved in metabolic pathways and mitotic division, respectively[48]. These exposures based on the MOAs give us a better understanding of how mechanistically bees may have increased susceptibility to other stressors like parasitic infections and how some exposure to fungicides and herbicides—which target highly conserved biological pathways—could have unintended, non-target effects on other beneficial biological organisms like honey bees.

### KEGG biological pathway analysis

When mapping the chemicals that have significant differences in terms of their relative abundances using the KEGG database pathways, we find new potential dysregulation in addition to what was previously identified using prior omic methods. Moreover, we have identified completely new metabolic pathways that may be affected (Fig 2). We are unable to determine the exact cause of the potential dysregulation and whether this is from the xenobiotic exposure or the *N*. *ceranae* infection itself, or another factor that has not been taken into account. However, we do know that the response to a number of stressors such as fungal and viral infections as well as pesticide exposure results in multiple changes of gene expression of metabolic pathways [33], supporting the notion that dysregulation of metabolism is likely playing a central role in the decline of bee health.

The most pervasive xenobiotic chemical that was found in all hives except one was naphthalene and overall there was a relative higher abundance found in *N*. *ceranae* infected hives. The pervasiveness of this chemical is perhaps not surprising as sources of naphthalene include vehicle, locomotive, and aircraft emissions, cooking, and residential wood combustion. In 2011, more than 527 tons of naphthalene was emitted in the New England area[49]. Naphthalene was previously used as an insecticide in bee hives to control beeswax moth, but is now banned for this application because it has been shown to accumulate in beeswax[50]. Here we show from the KEGG pathway analysis that consistent exposure to this is likely to disrupt the cytochrome p450 detoxification pathway (Fig 2B). This suggests that when bees are exposed to this at relatively high levels that other toxins from other xenobiotic exposure are likely to overwhelm the detoxification system, thereby causing more harm to the bee and resulting in higher sensitivity to sub-lethal exposures to a number of pesticides[51, 41–43].

We identified three chemicals (octadecanoic acid, (9Z)-octadecenoic acid, and hexadecanoic acid) associated with fatty acid metabolic pathways. Octadecanoic acid and (9Z)-octadecenoic acid are at the end of the fatty acid biosynthesis pathway and have relatively higher abundances in infected bees. These are known to be precursors of short chain fatty acids that have antibiotic properties when secreted by worker bees from their mandibular glands[52]. These fatty acids are also found in beebread[53], which suggests that infected worker bees could adaptively respond to infection by increasing the amount of antibiotics in bee bread that is fed to larval bees to prevent further transmission of a disease, as a general immune response. Hexadecanoic acid was found in relatively lower abundances, and according to the location within the KEGG pathway, it is a crucial link between fatty acid biosynthesis and degradation. This is important to note because these fatty acid pathways are known to play a role in immunocompetence [54–56].

The steroid biosynthesis pathway contained dysregulation of metabolites right next to each other along the same pathway, so this suggests that the enzyme that converts isofucosterol to beta-sitosterol, or vice versa, may be affected by the fungicide Fenpropimorph[57] (Fig 2B). Isofucosterol and beta-sitosterol are phytosterols, and derived forms are found in honey bee brood food and royal jelly. We speculate that brood feeding to worker and queen larvae by infected bees may be affected due to an imbalance of sterol production, and this may substantially impact the brood health as the major tissue sterol components of brood reared by the workers are known to be 24-methylenecholesterol, followed by sitosterol and isofucosterol[58].

Other lesser known metabolites affected include beta-tocopherol, which is part of the ubiquinone and other terpenoid-quinone biosynthesis pathway. Beta-tocopherol is a form of vitamin E and is also found in royal jelly. Supplements of this are known to increase royal jelly production in hives by worker bees, and the addition of this component to royal jelly is considered to play a key role in determining if an egg will develop into a queen bee[59]. Other related pathways affected include the terpenoid biosynthesis pathway, and squalene within this pathway has higher relative abundance in *N*. *ceranae* infected hives. Terpenoids including squalene are found in addition to hydrocarbons on the exoskeleton of bees, and changes in abundance of these have been shown to play a role in nest-mate recognition. However, it is more likely that the higher abundance observed is from the propolis that the forager bees collected as squalene is found in plants as well. Squalene has yet to be measured on the honey bee exoskeleton and is only found to play a role in nestmate recognition in stingless bees that have a coating of the collected plant resins on their exoskeleton[60]. We suspect the relative higher abundance is therefore most likely due to higher amounts of propolis being collected in the *N*. *ceranae* infected hives. Lastly, no direct connection could be drawn between the potential dysregulation of sulfur metabolism in honey bees and its impact, so further investigation on the implications of this potential dysregulation is needed.

### Potential interaction of xenobiotics and *N*. *ceranae*

We found many exposures that are potentially responsible for the disruption of various metabolic pathways and can interact with one another; this provides insight into how synergistic declines in bee health can occur. *N*. *ceranae* on its own interferes with nutrient acquisition[61]; therefore, other pesticide exposure effects can synergistically contribute to bee malnutrition, even when adequate pollen is available[31, 62]. Our results suggest that malnutrition is likely to arise from carbohydrate metabolic disruption due to a *N*. *ceranae* infection in combination with fatty acid and amino acid metabolism disruption from fungicide, insecticide, and herbicide exposure. The fungicide and herbicide identified in *N*. *ceranae* infected hives were Propamocarb and Carbetamide, respectively, which have multiple modes of action that may affect fatty acid metabolism as well as succinate dehydrogenase, which is assumed to be the target mode of action for many fungicides[48]. The fatty acid dysregulation may be compounded by the fact that honey bees are likely reliant upon a limited subset of fatty acid mobilization pathways when responding to energetic stress[63–65]. Instead, we found that the insecticide, Azobenzene, in *N*. *ceranae* infected hives, is a succinate dehydrogenase inhibitor[66]. Downstream of carbohydrate metabolism, succinate plays a role in ATP generation through the highly-conserved TCA cycle, and studies have shown ATP levels are impacted when bees are exposed to fungicide succinate dehydrogenase inhibitors[67]. Succinate is located upstream of amino acid metabolism so there is potential for amino acid metabolism disruption as well. *Nosema* infected bees are known to have significantly different amino acid composition[61], so this could be another potential metabolic pathway where synergistic effects resulting from multiple exposures could occur. Not only do we find histidine metabolism that could be potentially dysregulated at two different points, but we find that tyrosine metabolism could be potentially disrupted because there is a relatively higher abundance of 3,4-dihydroxyphenylethylene glycol located downstream of succinate.

On their own, sub-lethal exposures to fungicides and herbicides are safer for bees in comparison to insecticide exposures, but they have increased damaging effects when combined with other stressors such as insecticides and *N*. *ceranae* parasitic infections[68]. Our results suggest that the synergistic effects from all of these stressors could be the result from dysregulation of various points along multiple highly conserved metabolic pathways that are connected to one another through the TCA cycle.

### Conclusions

Both xenobiotic exposures and parasites such as *N*. *ceranae* are considered part of the measurable specific external exposome. This study demonstrates how xenobiotic exposures can potentially interact with diseases that might increase susceptibility to further infection, and it serves as a proof of concept for the integration of targeted disease screening with discovery-based exposomics using TOF mass spectrometry. This analytical platform measures and characterizes the effects of exposures from the specific external exposome (e.g., xenobiotics and *N*. *ceranae*) and from the internal exposome environment of the bees (metabolites). These data identify changes in the metabolome and provide a conduit for the identification of affected biological pathways. We postulate the changes in the internal environment of the bees result in a negative chemical feedback loop and further increase susceptibility to infection and, ultimately, the decline of hive health (Fig 1). In addition, through an integration of data, we can observe how xenobiotic exposures can possibly affect bee physiology through multiple modes of action, which might render bees more susceptible to pathogens like *N*. *ceranae*.

## Supporting information

S1 Fig Relative ion abundances of detected xenobiotics (log_2_ normalized for scaling). Relative ion abundances of detected xenobiotics (log_2_ normalized for scaling). Data is represented by medians and error bars represent interquartile ranges for each xenobiotic. For each category, there was no significant association between the relative level of exposure and *N*. *ceranae* infection load.
(DOCX)

S1 Table 2x2 contingency table. The case and controls groups did not vary significantly based on ion abundance greater than or less than the combined median ion abundance (24.37, Log_2_ normalized).
(DOCX)

S2 Table 2x2 contingency tables. A. Ion abundance greater than or less than the median ion abundance stratified by chemical category. B. 2x2 contingency tables. Ion abundance greater than or less than the median ion abundance stratified by chemical category.
(DOCX)

S3 Table Mode of action for the most common xenobiotics identified. (DOCX)

S4 Table Geography type and randomized sample identifiers. (DOCX)

S5 Table A list of primers used for sq-PCR analyses for *Nosema ceranae* and *Nosema apis*. MITOC-F, MITOC-R, APIS-F, APIS-R are taken from Hamiduzzaman, et al. (2010)[35] and RpS5-F, RpS5-R are taken from reference Thompson et al. (2007)[69].
(DOCX)

S1 Dataset Raw data of the study for public access. (XLSX)
