# Correction: Honey bee (*Apis mellifera*) exposomes and dysregulated metabolic pathways associated with *Nosema ceranae* infection

**DOI:** 10.1371/journal.pone.0215166

**Published:** 2019-04-04

**Authors:** Robert L. Broadrup, Christopher Mayack, Sassicaia J. Schick, Elizabeth J. Eppley, Helen K. White, Anthony Macherone

Fig 2 is incorrect. The authors have provided a corrected version here.

**Fig 2:**
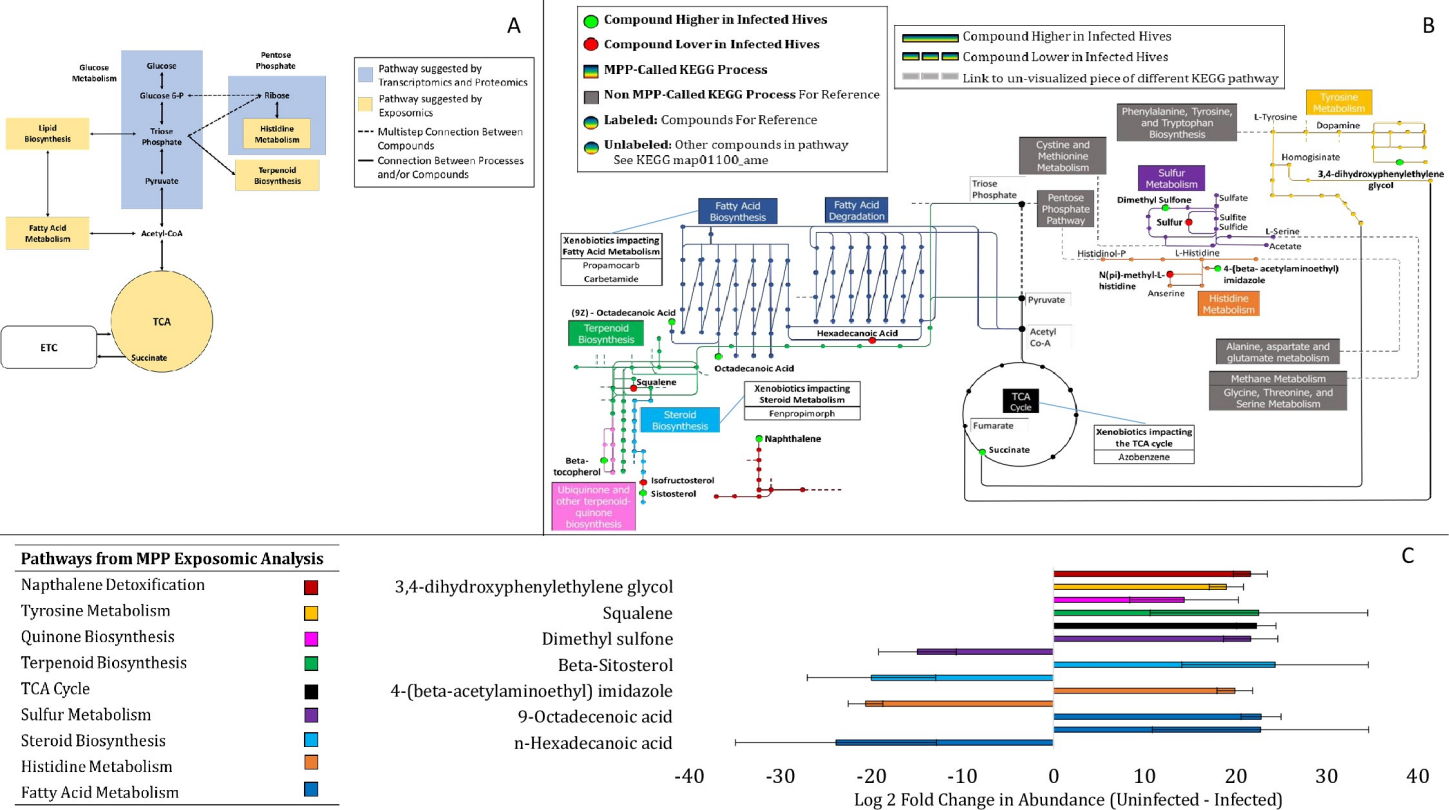
KEGG Pathway analysis. (A) Overview of affected metabolic pathways from a *N*. *ceranae* infection that have been determined from previous transcriptomic and proteomic analysis[30, 32, 33] and additional new pathways affected suggested by significant differences in metabolite relative abundances from the exposomic analysis. TCA represents The Citric Acid Cycle while the ETC represents the Electron Transport Chain. (B) Location of the 14 significantly higher or lower relative abundance compounds mapped onto 9 corresponding *Apis mellifera* metabolic pathways using the KEGG database. Compounds with significantly lower relative abundances are denoted with a red dot while chemicals with significantly higher abundances are denoted with a green dot. The pathways potentially affected are shown with different colors and their immediate connections revealed by comparing uninfected and uninfected hives are marked in red. Each color denotes a different metabolic pathway that contains a corresponding label. Other chemical names in the pathways are not labeled and less connected metabolic pathways have been removed for clarity. Grey boxes represent metabolic pathways not directly linked to a change in metabolite relative abundance in *N*. *ceranae* infected hives. (C) The difference of the means in fold changes (log 2) for the significant 14 chemical relative abundances identified between *N*. *ceranae* infected and uninfected hives corresponding to the nine KEGG pathways shown in Fig 2B. Error bars represent standard deviation of the difference between *N*. *ceranae* uninfected and infected chemical abundances.
