# Supplementary figures and images for: Honey bee (Apis mellifera) exposomes and dysregulated metabolic pathways associated with Nosema ceranae infection
Source: PLoS One. 2019 Mar 7;14(3):e0213249. doi: 10.1371/journal.pone.0213249 (PMC6405199; doi:10.1371/journal.pone.0213249)

**S1 Fig**


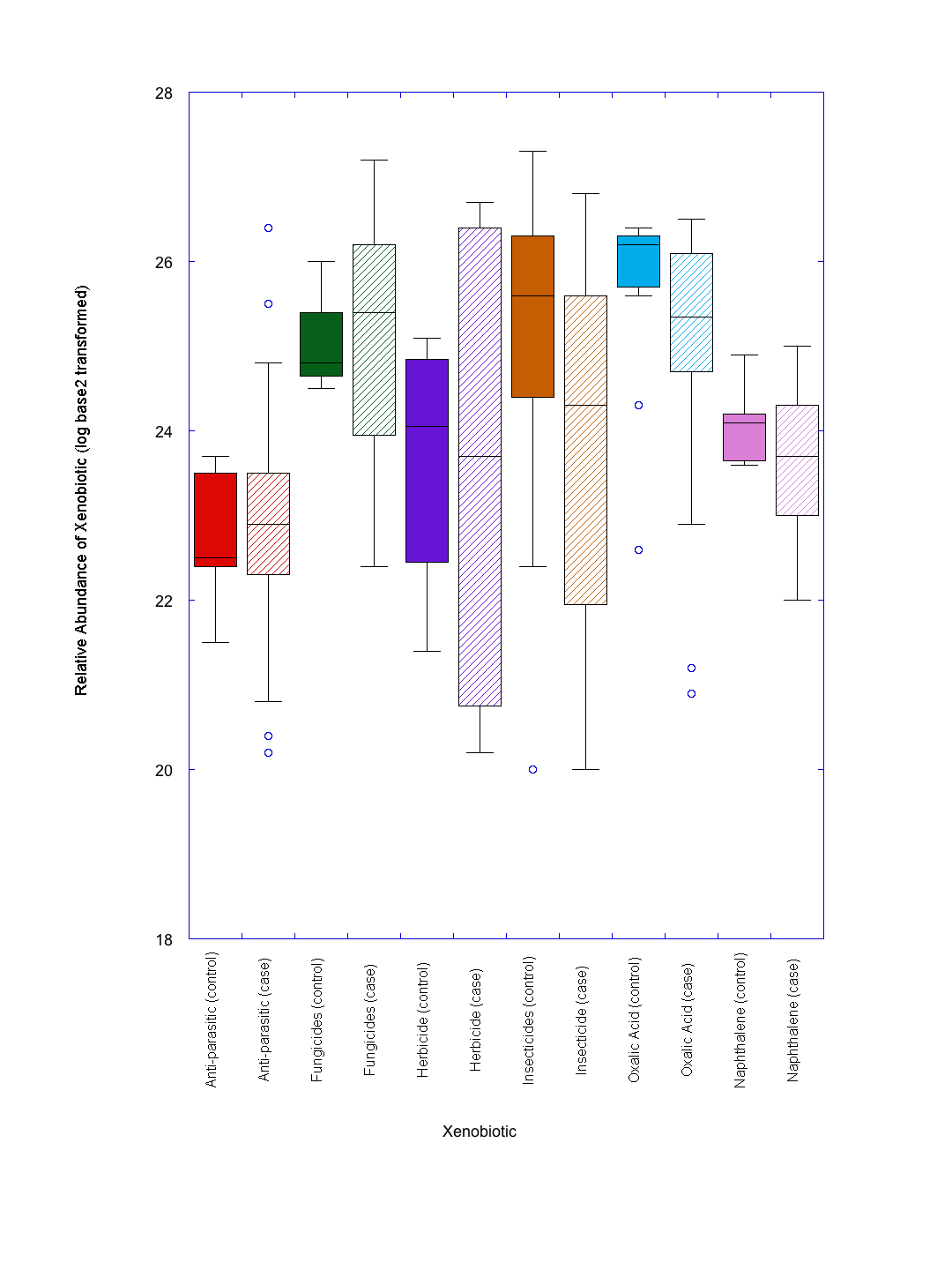

Supplement: S1 Fig — Relative ion abundances of detected xenobiotics (log2 normalized for scaling). Data is represented by medians and error bars represent interquartile ranges for each xenobiotic. For each category, there was no significant association between the relative level of exposure and N. ceranae infection load. (DOCX) [file pone.0213249.s001.docx]
