# Supplementary material for: Honey bee (Apis mellifera) exposomes and dysregulated metabolic pathways associated with Nosema ceranae infection
Source: PLoS One. 2019 Mar 7;14(3):e0213249. doi: 10.1371/journal.pone.0213249 (PMC6405199; doi:10.1371/journal.pone.0213249)
Supplement: S2 Table — A. Ion abundance greater than or less than the median ion abundance stratified by chemical category. B. 2x2 contingency tables. Ion abundance greater than or less than the median ion abundance stratified by chemical category. (DOCX) [file pone.0213249.s003.docx]

**S2 Table**

A. Ion abundance greater than or less than the median ion abundance stratified by chemical category.

B. 2x2 contingency tables. Ion abundance greater than or less than the median ion abundance stratified by chemical category.
