# Supplementary material for: Honey bee (Apis mellifera) exposomes and dysregulated metabolic pathways associated with Nosema ceranae infection
Source: PLoS One. 2019 Mar 7;14(3):e0213249. doi: 10.1371/journal.pone.0213249 (PMC6405199; doi:10.1371/journal.pone.0213249)
Supplement: S4 Table — (DOCX) [file pone.0213249.s005.docx]

**S4 Table**
