# Supplementary material for: Honey bee (Apis mellifera) exposomes and dysregulated metabolic pathways associated with Nosema ceranae infection
Source: PLoS One. 2019 Mar 7;14(3):e0213249. doi: 10.1371/journal.pone.0213249 (PMC6405199; doi:10.1371/journal.pone.0213249)
Supplement: S5 Table — MITOC-F, MITOC-R, APIS-F, APIS-R are taken from Hamiduzzaman, et al. (2010)[35] and RpS5-F, RpS5-R are taken from reference Thompson et al. (2007)[69]. (DOCX) [file pone.0213249.s006.docx]

**S5 Table**
